# Thymic Versus Induced Regulatory T Cells – Who Regulates the Regulators?

**DOI:** 10.3389/fimmu.2013.00169

**Published:** 2013-06-27

**Authors:** Giovanni Antonio Maria Povoleri, Cristiano Scottà, Estefania Andrea Nova-Lamperti, Susan John, Giovanna Lombardi, Behdad Afzali

**Affiliations:** ^1^Medical Research Council Centre for Transplantation, King’s College London, London, UK; ^2^National Institute for Health Research Biomedical Research Centre at Guy’s and St Thomas’ NHS Foundation Trust and King’s College London, London, UK; ^3^Department of Immunobiology, King’s College London, London, UK

**Keywords:** regulatory T cells, Foxp3, Tr1, Th3, iTr35, interleukin-17, epigenetics, microRNA

## Abstract

Physiological health must balance immunological responsiveness against foreign pathogens with tolerance toward self-components and commensals. Disruption of this balance causes autoimmune diseases/chronic inflammation, in case of excessive immune responses, and persistent infection/immunodeficiency if regulatory components are overactive. This homeostasis occurs at two different levels: at a resting state to prevent autoimmune disease, as autoreactive effector T-cells (Teffs) are only partially deleted in the thymus, and during inflammation to prevent excessive tissue injury, contract the immune response, and enable tissue repair. Adaptive immune cells with regulatory function (“regulatory T-cells”) are essential to control Teffs. Two sets of regulatory T cell are required to achieve the desired control: those emerging *de novo* from embryonic/neonatal thymus (“thymic” or tTregs), whose function is to control autoreactive Teffs to prevent autoimmune diseases, and those induced in the periphery (“peripheral” or pTregs) to acquire regulatory phenotype in response to pathogens/inflammation. The differentiation mechanisms of these cells determine their commitment to lineage and plasticity toward other phenotypes. tTregs, expressing high levels of IL-2 receptor alpha chain (CD25), and the transcription factor Foxp3, are the most important, since mutations or deletions in these genes cause fatal autoimmune diseases in both mice and men. In the periphery, instead, Foxp3^+^ pTregs can be induced from naïve precursors in response to environmental signals. Here, we discuss molecular signatures and induction processes, mechanisms and sites of action, lineage stability, and differentiating characteristics of both Foxp3^+^ and Foxp3^−^ populations of regulatory T cells, derived from the thymus or induced peripherally. We relate these predicates to programs of cell-based therapy for the treatment of autoimmune diseases and induction of tolerance to transplants.

## Introduction

**Figure 1:**
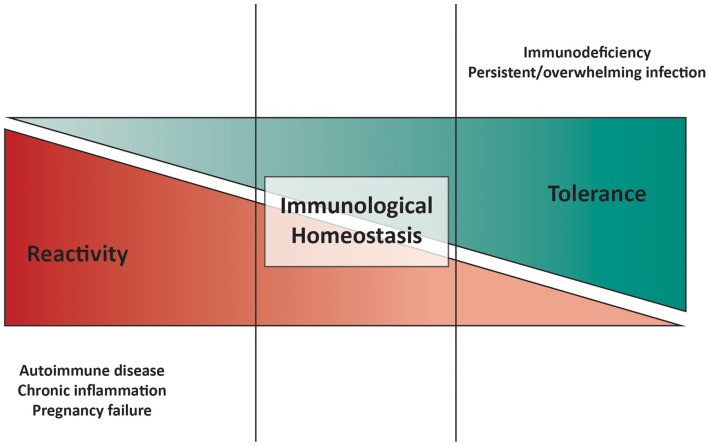
**Model of immunological homeostasis**. Disturbance of the balance between pro-inflammatory and anti-inflammatory mechanisms is shown at either end of the cartoon. On the one hand, excessive immune responsiveness and/or deficiency in tolerogenic mechanisms can lead to autoimmune diseases, chronic inflammation, and pregnancy failure. On the other hand, weak immune responsiveness and/or excessive tolerance-inducing machineries can result in in immunodeficiency, characterized by recurrent, and/or overwhelming infections.

Physiological health requires a balance between immunological responsiveness against foreign pathogens and tolerance toward self-components and commensals. The immune system must guarantee this homeostatic balance, since its disruption leads to autoimmune diseases (AID) and chronic inflammation in the event of excessive immune reactivity, on the one hand, and persistent infection(s) and immunodeficiency on the other (Figure 1).

Regulation of immune responses occurs concurrently at two different levels: in the “pathogen-free” environment (where “danger” is inherently internal), to maintain tolerance to self-components, and in the “pathogen-containing” environment (where “danger” is external), to prevent excessive tissue injury, contract the immune response and enable tissue repair.

Central selection of the T cell repertoire imparts intrinsic autoreactivity to adaptive immunity as only T cells capable of recognizing self-MHC are positively selected for survival. Thus, despite negative selection of strongly autoreactive thymocytes, the mature immune system can clearly be demonstrated to contain T cells with autospecificity (Muraro et al., 1997), necessitating active regulation of these cells in the periphery. That autoreactive cells exist in the neonatal circulation imparts an obligate requirement for the presence of regulation from birth, a function ascribed to non-redundant “thymically derived” regulatory T cells (tTregs).

Although tTregs have been the focus of the “immunoregulation” literature in recent years, the adult T cell pool also contains a series of other T cells with regulatory function, many of which are induced to develop suppressive phenotypes in the periphery in response to antigenic challenges and the local micro-environment. Such “induced” regulatory T cells include induced Foxp3^+^ (iTregs) and Foxp3^−^ (Th3, Tr1, iTr35, and CD8^+^CD28^−^) populations of cells.

In this review, we describe the origins and functions of different T cells with regulatory function, detailing their properties. An important note that is worth highlighting at the outset is one of semantics. In this review, we refer to CD4^+^CD25^hi^FoxP3^+^ regulatory T cells as “Tregs.” As there have been recent calls for greater clarity in the nomenclature of FoxP3^+^ regulatory T cells (Abbas et al., 2013) (Table 1), we refer to thymically derived Tregs as tTregs and peripherally derived Tregs as pTregs. All *in vitro* induced FoxP3^+^ Tregs we will call iTregs. All other inducible regulatory T cell populations will be referred to by their current internationally accepted names, such as Tr1 cells.

**Table 1:** **Recommendations for Treg cell nomenclature**.

“Thymus-derived Treg cell (tTreg cell)” should be used instead of “natural Treg cell (nTreg cell)”
“Peripherally derived Treg cell (pTreg cell)” should be used instead of “induced or adaptive Treg cell (iTreg cell or aTreg cell)”
“*In vitro* induced Treg cell (iTreg cell)” should be used to clearly distinguish between those Treg cell populations generated *in vivo* versus those generated *in vitro*
Treg cell terms should be used only when there is definitive evidence justifying their use
The development and use of new Treg cell terminology should be limited, especially for subpopulations

*Reproduced from Abbas et al. (2013)*.

## Foxp3^+^ Regulatory T Cells

The relative importance of centrally derived tolerance-inducing T cells was established by experiments between the late 1960s and early 1980s where it was observed that thymectomy of mice on the third day of life resulted in organ-specific autoimmune diseases [the exact target organ(s) depending on the mouse strain used]. However, this did not occur if neonatal mice were thymectomized on days 1 or 7 (Nishizuka and Sakakura, 1969; Kojima et al., 1976, 1980; Taguchi and Nishizuka, 1981) and day 3 thymectomized mice would not develop autoimmunity after infusion of thymocytes (Sakaguchi et al., 1982). These experiments suggested that autoreactive T cells exit the thymus in the first 3 days of life followed a few days later by a population of suppressor cells that control the autoreactive cohort. These experiments were followed by the first descriptions of Tregs by Sakaguchi et al. (1995, 1996) as a circulating subset of CD4^+^ T cells expressing high levels of CD25 (the IL-2 receptor α-chain), which could prevent the development of multi-organ autoimmune diseases (thyroiditis, gastritis, insulitis, sialoadenitis, adrenalitis, oophoritis, glomerulonephritis, and polyarthritis) and/or rodent graft-versus-host disease (GVHD)-like wasting disease in thymectomized mice, by adoptive transfer (Suri-Payer et al., 1998). This was an advance on previous observations that had identified the “rescuing” population as Thy1^+^(CD90^+^) Lyt1^+^(CD5^+^) Lyt2^−^ (CD8a^−^) Lyt3^−^(CD8b^−^) (Sakaguchi et al., 1982) CD45RB^lo^ (Morrissey et al., 1993). As CD25 correlates positively with CD5 and negatively with CD45RB, the identification of CD25 expression as a surface marker for Tregs was biologically plausible. The subsequent identification of humans and mice deficient in CD4^+^CD25^hi^ cells (as a result of mutations in the *FOXP3* and *Foxp3* genes respectively – see below), which develop severe autoimmune diseases (Sakaguchi et al., 1995, 1996; Chatila et al., 2000; Wildin et al., 2001) strongly suggests that these cells have a critical and non-redundant regulatory role in the maintenance of self-tolerance.

Although CD25 expression was the original defining feature of Tregs, CD25 is also expressed by antigen-experienced and recently activated conventional T cells with non-regulatory properties (effector T cells, “Teff”). As a result, CD25 is of greatest sensitivity when used to identify Tregs from naïve T cell populations, such as human umbilical cord blood, or antigen-naïve animals. Thus, in antigen-experienced mammals, only the top 2–5% of CD25 expressing CD4^+^ cells (CD25,^hi^) contains genuine Tregs (Baecher-Allan et al., 2001). Since the descriptions of Tregs, therefore, a number of additional markers have been proposed as Treg-specifying, including cytotoxic T-lymphocyte antigen-4 (CTLA-4) (Wing et al., 2008; Sakaguchi et al., 2009), GITR (glucocorticoid-induced TNF receptor family related protein; TNFRSF18) (Shimizu et al., 2002), CD39 (Deaglio et al., 2007), HLA-DR (Baecher-Allan and Hafler, 2006), CD45RA (Miyara et al., 2009), and low expression of CD127 (the IL-7 receptor α-chain) (Liu et al., 2006). While these markers will not be the focus of this review, it is important to note that none can be used as unambiguous identifiers of human Tregs; however, they often identify subsets of Tregs with different (quantities or mechanisms of) suppressive functions, implying that there is considerable heterogeneity in human populations of Tregs. Such heterogeneity and the lack of specific markers for the Treg lineage remain the cornerstone of debates regarding whether Tregs are in fact a distinct T cell lineage and/or a possibility in the life cycle of many different T cells.

### Forkhead box P3, the key transcription factor of Tregs

The Scurfy mouse (*sf*), an X-linked mutant strain, described in 1949 [cit. loc (Russell et al., 1959)], exhibits a series of autoimmune features including skin scaliness, diarrhea, and death (between 2 and 4 weeks after birth) in association with CD4^+^ T cell hyper-proliferation, multi-organ CD4^+^ cell infiltration (Blair et al., 1994) and over-production of several inflammatory cytokines (Kanangat et al., 1996). This fatal autoimmune lymphoproliferative syndrome was found to map to a gene locus on the X chromosome called *Foxp3*, which was described as a new member of the forkhead/winged-helix family of transcription factors (TF) (Brunkow et al., 2001). The *Foxp3* gene is highly conserved between species and a mutation in the human gene, *FOXP3*, was identified as the causative factor responsible for the human equivalent of Scurfy, the Immunodysregulation, Polyendocrinopathy, and Enteropathy, X-linked syndrome (IPEX), also known as X-linked autoimmunity and allergic dysregulation syndrome (XLAAD) (Chatila et al., 2000; Bennett et al., 2001; Hori et al., 2003). Both mouse and human diseases have deficient circulating Tregs, which suggests that *Foxp3* and *FOXP3* are essential for normal Treg development in the two species respectively. This position is strengthened by the failure of *Foxp3* knockout mice to develop circulating Tregs; these animals develop a Scurfy-like syndrome from which they can be rescued by the adoptive transfer of Tregs from a *Foxp3* replete animal (Fontenot et al., 2003). Furthermore, ectopic or over-expression of *Foxp3* in CD4^+^CD25^−^ mouse cells results in the development of a Treg phenotype (Fontenot et al., 2003; Hori et al., 2003; Khattri et al., 2003). In mice, Foxp3 expression is a good phenotypic marker of Tregs (Fontenot et al., 2005c; Wan and Flavell, 2005); in humans, however, FOXP3 does not allow the unambiguous identification of Tregs (Ziegler, 2006) as it is induced during TCR stimulation in conventional CD4^+^ T cells (Walker et al., 2003; Gavin et al., 2006; Wang et al., 2007) (in much the same manner as CD25) and there has been some debate as to whether the induced CD4^+^CD25^+^FOXP3^+^ population is suppressive or anergic (Walker et al., 2003; Gavin et al., 2006).

Although Foxp3 may function as a transcriptional inhibitor through associations with NFAT, NF-κB, and RORγt (Schubert et al., 2001; Bettelli et al., 2005; Zhou et al., 2008a), its biological function is still incompletely understood and will be discussed in an accompanying review in this series. However, it is worth mentioning that the concept of Foxp3 as a “lineage-specifying factor” of Tregs is an over-simplification, as suggested by three lines of evidence: (i) Foxp3 is not sufficient in itself to determine the full Treg transcriptional profile (Hill et al., 2007); (ii) Foxp3 is expressed by (human) Teffs following activation, without imparting the phenotype associated with Tregs; (iii) humans with IPEX syndrome have heterogeneous T cell abnormalities, including dysfunction in Teffs (Bacchetta et al., 2006).

### Thymically derived Tregs

Thymic education of T cells is a two step process involving, first, positive selection of thymocytes recognizing self-MHC and, second, negative selection of T cells with T cell receptors (TCRs) of high avidity for class I and class II MHC molecules presenting self-antigens. Thus, duration and avidity of the TCR interaction with self-peptide-MHC complexes on antigen-presenting cells (APC) determine thymocyte fate. Thymocytes that bind with high avidity undergo programed cell death in an attempt to limit autoreactivity in the periphery, while thymocytes with low avidity for self-MHC:peptide are selected as effector T cells (Teff).

A thymic origin for Tregs was suggested by the neonatal thymectomy-induced autoimmunity models described above (reviewed in Shevach, 2000). In addition, neonatal infection of BALB/c mice with superantigen-expressing murine mammary virus (MMV) results in increased numbers of Vβ6^+^ Tregs (Papiernik et al., 1998), which implies that thymocyte interaction with antigen preferentially favors Treg differentiation. Indeed, interactions between TCR and MHC class II peptides are essential for normal tTreg development (Sakaguchi et al., 2008; Josefowicz and Rudensky, 2009), an assertion which is consistent with the observation that Tregs express molecules associated with an activated state in Teffs (CD5, CD25, CTLA-4, and Foxp3) and the binding of TCR/CD28-coupled TFs (e.g., NFAT and AP1) to the *Foxp3* promoter (Mantel et al., 2006). Thus, mice engineered for high antigen expression, e.g., influenza haemaglutinin (HA), and TCR specificity for that HA (i.e., I-E^d^-restricted TCR specific for HA) develop large numbers of Tregs (Jordan et al., 2001), indicating that self-agonist ligands, contrary to inducing clonal deletion, or anergy, cause central development of Tregs. These observations are corroborated by a high degree of self-reactivity (against MHC/peptide complexes expressed on APCs) in Tregs compared to other CD4^+^ populations (Romagnoli et al., 2002). This demonstrates a biased thymically imprinted TCR repertoire based on recognition of self-MHC-peptide, suggesting that negative selection in the thymus is incomplete, with thymocytes having TCR-MHC:self-peptide interactions of intermediate strength escaping deletion and differentiating into cells with a regulatory phenotype (Tregs) (reviewed in Singer et al., 2008; Josefowicz et al., 2012). There is now significant evidence that tTreg development is self-antigen driven, with the tTreg population being largely autoreactive (Hsieh et al., 2004, 2006; Picca and Caton, 2005). The high similarity between the TCR repertoire of Tregs found within the thymus and Tregs isolated from the circulation (Hsieh et al., 2006; Wong et al., 2007) is, therefore, indicative of thymic Treg émigrés making a significant contribution to the peripheral Treg pool.

A number of additional cues are required for thymic induction of Tregs, notably those providing co-stimulation or IL-2R-γ_c_ cytokine family signaling. The importance of γ_c_ cytokines to tTreg development is highlighted by the absence of this population from the thymus and periphery of IL-2R-γ_c_ knockout animals (Fontenot et al., 2005b) and spontaneous development of autoimmune diseases in mice lacking IL-2Rβ (CD122), which can be prevented by infusion of donor Tregs (Suzuki et al., 1995; Malek et al., 2002). Although no single member of this cytokine family (IL-2, IL-7, or IL-15) is non-redundant in the thymic induction of tTregs, the most significant defect is observed in IL-2^−*/*−^ or CD25^−*/*−^ animals, in which Foxp3 expression is reduced by 50% in thymocytes and animals succumb to lethal autoimmune diseases (Sadlack et al., 1993; Willerford et al., 1995; Fontenot et al., 2005b). IL-2, the most important γ_c_ family member for tTreg induction (Fontenot et al., 2003; Hori et al., 2003) activates Stat5 through γ_c_ chain-associated Janus Kinase (JAK) 3; pY-Stat5 subsequently binds to the promoter region of *Foxp3* to positively regulate the gene (Zorn et al., 2006; Burchill et al., 2007; Yao et al., 2007). As expected, *Jak3*^−*/*−^ and *Stat5*^−*/*−^ mice have few or no circulating Foxp3^+^ cells (Mayack and Berg, 2006; Yao et al., 2007). Of note, developing Treg-precursors in the thymus are highly attuned to IL-2 as they express CD25 and thus have a competitive advantage in the IL-2-poor environment of the thymus. Thus, even suboptimal IL-2Rβ signaling, for example through mutations of Y → F (tyrosine to phenylalanine) at key sites binding Shc or Stat5, is sufficient to support normal tTreg (but not iTreg) development (Yu et al., 2009; Cheng et al., 2013).

Co-stimulation through CD28 is particularly important for tTreg development as both CD80/CD86 and CD28 knockout animals (Salomon et al., 2000; Tai et al., 2005) have striking tTreg deficiency. Signals transduced through the TCR and CD28 that are clearly important in thymic Treg lineage commitment include both the NF-κB and Ras-Raf-MAPK pathways. This is demonstrated through inhibition of tTreg development by disruptions to components of either the NF-κB, e.g. Bcl10, PKCθ, CARMA1, IκB kinase 2, c-Rel, TRAF6 (Schmidt-Supprian et al., 2003, 2004; Barnes et al., 2009; Isomura et al., 2009; Long et al., 2009; Grigoriadis et al., 2011; Shimo et al., 2011; Schuster et al., 2012), or the Ras-Raf-MAPK pathways, such as RasGRP1 and Raf (Willoughby et al., 2007; Chen et al., 2008).

In contrast to previous reports suggesting that TGF-β is not required for the thymic induction of thymocytes (Marie et al., 2005; Li et al., 2006), conditional ablation of TGF-βRI in double-positive (CD4^+^CD8^+^; DP) thymocytes does result in a temporary reduction of Foxp3^+^ thymocytes in neonatal mice (Liu et al., 2008), suggesting that central Treg selection may be enhanced by TGF-β signaling. In contrast, Akt signaling in developing thymocytes suppresses Treg development through mTOR (Haxhinasto et al., 2008), in a manner akin to iTregs (see below).

These observations are consistent with a step-wise model (Burchill et al., 2008; Lio and Hsieh, 2008) in which Tregs are selected from late-stage, single-positive (CD4^+^) thymocytes (Fontenot et al., 2005a), whose TCRs engage high affinity ligands (Sakaguchi et al., 2008) presented by either medullary or cortical thymic epithelial cells (mTECs or cTECs) in the context of MHC class II (Aschenbrenner et al., 2007; Liston et al., 2008b) and in the presence of CD28 co-stimulation (Tai et al., 2005). Thus, TCR/CD28 engagement induces expression of CD25 by thymocytes, sensitizing them to IL-2, which instructs Foxp3 and CD25 expression in a Stat5-regulated manner (Burchill et al., 2008; Lio and Hsieh, 2008). However, there is also some evidence that Tregs may, in fact, be induced to differentiate at a much earlier, double-positive (CD4^+^CD8^+^), developmental stage before agonist selection (Pennington et al., 2006). This is consistent with demonstrations in K14-A_β_^b^ mice that, similar to other CD4^+^ T cells, positive selection on thymic cortical epithelium is sufficient for Treg differentiation from DP precursors (Bensinger et al., 2001).

### Peripherally induced Tregs

**Figure 2:**
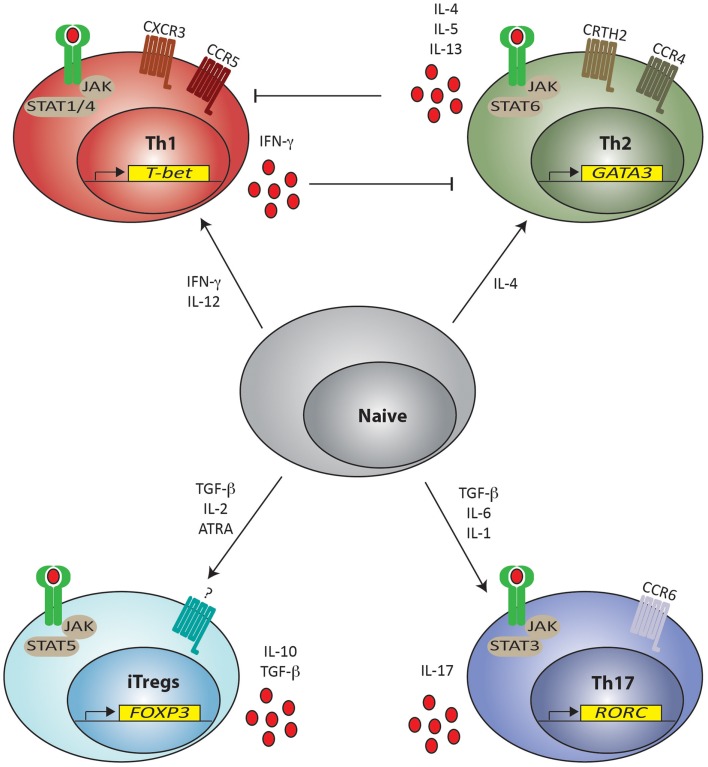
**Naïve T helper cell differentiation pathways for lineage commitment in the periphery**. T helper cells (Th) can be induced from naïve CD4^+^ cells to differentiate toward Th1, Th2, Th17 and Treg phenotypes depending on the cytokine milieu present in the environment. Presence of IFN-γ and IL-12 promote skewing toward Th1 commitment by signaling through STAT1 and STAT4, respectively. Th1 cells are characterized by expression of *T-bet*, chemokine receptors CCR5 and CXCR3 and produce IFN-γ, which inhibit Th2 differentiation. Th2 cell commitment is instead promoted by IL-4 via STAT6 signaling. Th2 committed cells express *GATA-3*, chemokine receptors CCR4 and CRTH2 and secrete IL-4, IL-5, and IL-13, which in turn inhibit Th1 differentiation. Development of both iTreg and Th17 phenotypes requires the presence of TGF-β, but the proinflammatory IL-6 and IL-1 preferentially skews the response toward a Th17 phenotype through STAT3 signaling leading to the expression of *RORC*. Th17 cells express the chemokine receptor CCR6 and secrete IL-17. iTregs are instead induced in the presence of TGF-β together with IL-2 (and ATRA) through STAT5 signaling, leading to the expression of *Foxp3* and can secrete IL-10 and TGF-β. A definite chemokine receptor for iTregs has not yet been clearly described. JAK = Janus kinase; STAT = Signal Transducer and Activator of Transcription. ATRA = all-trans retinoic acid.

There is also significant evidence showing that, like other CD4^+^ lineages, Tregs can be generated from CD4^+^ naïve precursors in the periphery. Here, host detection of infection and tissue injury initiates events that result in recruitment and differentiation of CD4^+^ T helper (Th) lymphocytes to functions suited to removal/containment of the noxious stimulus. Specific signaling pathways essential for differentiation, expression of key TFs, specific cytokines, and surface molecules distinguish distinct CD4^+^ Th lineages from each other. Thus, pluripotent naïve CD4^+^ T cells (Thp) are induced to “commit” to particular lineages by mode of stimulation, antigen concentration, co-stimulation, and cytokine milieu (Constant and Bottomly, 1997) through distinct pathways, including, but not exclusively, Stat1/Stat4 (Th1), Stat6 (Th2), Stat5 (Treg), and Stat3 (Th17) (Zhu et al., 2010). Each lineage is then characterized by expression of its own cytokine profile: IFN-γ (Th1), IL-4 (Th2), and IL-17 (Th17); dominant TFs: T-bet (Th1), Gata-3 (Th2), Foxp3 (Treg), and Rorc (Th17) (Zheng and Flavell, 1997; Szabo et al., 2000, 2002; Fontenot et al., 2003; Wan and Flavell, 2005; Ivanov et al., 2006) and chemokine receptors: CCR5 and CXCR3 (Th1), CRTH2 and CCR4 (Th2), and CCR6 (Th17) (Figure 2). Individual lineages are specialized to promote specific biological functions, for example, immunity against intracellular microorganisms (Th1), humoral immunity to control helminthic and other extracellular pathogens (Th2), clearance of extracellular bacteria, and fungi at mucosal surfaces (Th17) and regulation of immune system activation (Tregs) (Zhou et al., 2009a; Zhu et al., 2010). Thus, detection of “danger” is a key event in the initiation of this cascade and recruitment and differentiation of the most appropriate Th lineage(s) is the key determinant of pathogen removal/persistence and tissue repair/healing during immune responses.

The conditions favoring peripheral induction of Tregs (pTregs) include suboptimal dendritic cell (DC) activation, sub-immunogenic doses of agonist peptide, mucosal administration of peptide and presence of appropriate cytokines, notably TGF-β and IL-2 (Chen et al., 2003; Apostolou and von Boehmer, 2004; Kretschmer et al., 2005; Selvaraj and Geiger, 2007; Siewert et al., 2008; Zheng et al., 2008). The greater the strength of the TCR/MHC:peptide interaction and co-stimulation, the greater the requirement for tolerance-inducing cytokines, specifically TGF-β and IL-2, to induce a regulatory, as opposed to, effector phenotype in Thp. Some of this effect is related to the ability of high concentrations of TGF-β to down-regulate receptors for other cytokines, including IL-6 (Zheng et al., 2008), and the ability of IL-2-activated Stat5 to inhibit loci of other lineages (Laurence et al., 2007), implying that efficient pTreg differentiation is at least partially contingent on inhibition of differentiation to alternate Th lineages. This assertion is supported by evidence that the presence of cytokines required for T cell skewing to alternate Th lineages, such as IL-12 (to Th1) and IL-6 (to Th17) (Figure 2) preferentially foster development of those lineages in contrast to iTreg through induction of lineage-specifying Stat proteins and TFs (Wei et al., 2007).

Of particular note, it appears that not all Thp can differentiate in the periphery to Tregs (Hsieh et al., 2004; Lathrop et al., 2008). Instead, recent evidence indicates that either the thymus may remain a site of Treg differentiation during immune responses (Zelenay et al., 2010) or that recent thymic émigrés are, in fact, the precursors of pTregs (Paiva et al., 2013). Speculatively, the implication is that either T cells with certain TCR specificities are more suited to differentiate into Tregs (presumably due to higher than average TCR avidity for self-MHC:peptide) or that signals received by pTreg-precursors in the thymus ensure that the *Foxp3* locus is epigenetically in a state ready for gene transcription in the periphery. Nevertheless, pTregs suppress antigen driven CD4^+^ T-cell expansion and both Th1 and Th2 cytokine production *in vitro* in a manner akin to tTregs (Chen et al., 2003); the paucity of distinguishing phenotypic and functional characteristics between tTregs and pTregs is one argument for a high degree of similarity in the signals required for their induction.

The requirement for low level TCR signaling for induction of pTregs is highlighted by experiments in which *in vivo* Foxp3 induction in Thp inversely correlates with the dose of immunogen (Kretschmer et al., 2005) and in which augmenting TCR signaling by removing an inhibitory E3 ubiquitin ligase (Chiang et al., 2000) inhibits Foxp3 induction (Wohlfert et al., 2006). Consistent with this, *in vitro* iTreg induction is inhibited by increasing concentrations of activating anti-CD3 (Kim and Rudensky, 2006) whereas premature termination of TCR signaling soon after T cell activation or inhibition of the PI3 Kinase/Akt/mTOR pathway downstream of TCR signaling augments iTreg induction (Sauer et al., 2008). Similarly, while CD28 signaling is critical for central selection of Tregs (Salomon et al., 2000), peripheral pTreg induction is, in contrast, inhibited by strong CD28 ligation (Kim and Rudensky, 2006; Benson et al., 2007), which explains why mice deficient in CTLA-4, an inhibitor of T cell activation, have impaired pTreg induction (Zheng et al., 2006). Similarly, anaphylatoxin receptor signaling activates the mTOR pathway; thus *C3ar1*^−*/*−^ or *C5ar1*^−*/*−^ mice have impaired mTOR signaling and take on an iTreg phenotype in response to TGF-β more readily than wild-type T cells. Antagonism of C3aR and C5aR in human naïve CD4^+^ T cells induces functional iTregs (Strainic et al., 2012).

TGF-β directly regulates the *Foxp3* gene through both TGF-β-inducible early gene 1 (TIEG1) and Mothers Against Decapentaplegic 3 (Smad3), which bind at promoter and enhancer regions in the *Foxp3* gene to upregulate its expression (Tone et al., 2008; Venuprasad et al., 2008) (see below). Notch-pathway mediated signals synergize with TGF-β to enhance Foxp3 expression by recruiting Notch1, CSL, and Smad proteins to promoter regions of the *Foxp3* gene (Samon et al., 2008). *In vivo*, DC populations producing local TGF-β are clearly sufficient to induce iTregs (Benson et al., 2007; Yamazaki et al., 2008).

The presence of all-trans retinoic acid (ATRA) in the Thp environment synergizes with TGF-β to promote iTreg development; this effect is sufficient to allow iTreg development even in the presence of high levels of co-stimulation (Benson et al., 2007). While receptor-ligand-mediated gene transcription is retinoic acid receptor (RAR)-α dependent (Elias et al., 2007; Hill et al., 2008), ATRA promotes iTreg differentiation both directly, through inhibition of differentiation to alternative lineages, notably Th17 (Elias et al., 2007; Mucida et al., 2007; Xiao et al., 2008), and indirectly, through the inhibition of environmental cytokines produced by CD44^hi^ effector memory T cells, especially IL-4 and IFN-γ (Hill et al., 2008), which support the development of alternative Th lineages. ATRA, moreover, imprints a gut-homing phenotype on iTregs (α4β7^+^CCR9^+^) (Benson et al., 2007). This is noteworthy as CD103^+^CD11c^+^ DCs present in lamina propria of small and large bowel, mesenteric lymph nodes, and Peyer’s patches induce an identical gut-homing phenotype (Annacker et al., 2005; Johansson-Lindbom et al., 2005) and the development of iTregs through secretion of local TGF-β and ATRA (Coombes et al., 2007; Sun et al., 2007). Such local milieu for the induction of iTregs might reflect the need to control immune responses directed against antigens expressed by local microbiota and ingested food and may provide an evolutionary link between iTregs and commensal bacteria. This may explain why several studies have reported a reduction in lamina propria Tregs of mice housed in germ-free environments (Strauch et al., 2005; Östman et al., 2006; Ishikawa et al., 2008), which may be related to the specific organisms that are present or absent from the “germ-free” environment (Ivanov et al., 2008).

### Regulation of *Foxp3* gene expression

**Figure 3:**
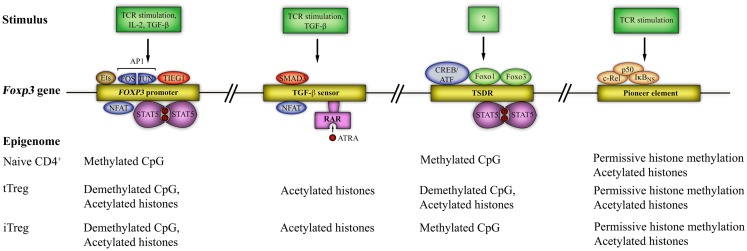
**The *Foxp3* epigenome and transcription factor binding sites**. Four distinct regions of the *Foxp3* gene are susceptible to epigenetic modification. These are the *Foxp3* promoter and three other conserved non-coding sequences (CNS): CNS1 – the TGF-β sensor/enhancer, CNS2 – Treg-cell-specific demethylation region (TSDR), and CNS3 – a *Foxp3* pioneer element. Epigenetic modifications include histone acetylation/deacetylation and CpG methylation/demethylation and are shown at each locus. Known transcription factor binding sites and the signals required for access to each region are also shown. STAT5 = signal transducer and activator of transcription 5, SMAD3 = small body size mothers against decapentaplegic 3, CREB = cAMP-response-element binding protein, ATF = activating transcription factor, NFAT = nuclear factor of activated T cells, AP1 = activator protein 1 (a dimer of FOS and JUN), TIEG1 = TGF-β-inducible early gene 1; Ets = E-twenty six; RAR = retinoic acid receptor; ATRA = all-trans retinoic acid; Foxo = forkhead box o.

Epigenetic mechanisms, such as DNA methylation, histone modification, nucleosome positioning, as well as microRNAs (miRNAs), are essential for control of gene expression (Baltimore et al., 2008; Wilson et al., 2009; Cedar and Bergman, 2011). Chromatin remodeling has a role in determining the accessibility of genes by transcriptional activators or repressors. In particular, methylated DNA sequences are “silenced”, while opening of the locus for transcription is linked to demethylation. For comprehensive reviews, the reader is referred to (Wilson et al., 2009; Cedar and Bergman, 2011). *Foxp3* gene expression is controlled by four elements, containing conserved non-coding sequences (CNS). The first is in the promoter region, two are in the first intron (CNS1 and CNS2, at 2 and 4.5 kb downstream of the transcriptional start site (TSS) of murine *Foxp3*, respectively) and the fourth (CNS3, at 7 kb downstream of the TSS of murine *Foxp3*) is in the second intron. These sites are regulated by epigenetic modifications that determine chromatin structure and DNA methylation, altering the accessibility of the gene locus to TFs. Known TF binding and epigenetic modifications at these sites are shown in Figure 3.

Three important caveats should be noted here. The first is that emerging evidence suggests a role for Foxp3 binding within enhancer elements in the *Foxp3* gene, exploiting enhancers “established” by Foxp3 predecessors, such as Foxo1 (Samstein et al., 2012). These data are not included in Figure 3. The second is that Foxp3 expression alone is insufficient for establishment of the Treg lineage; rather, the development of a Treg-specific genome-wide methylation pattern (“nTreg-Me”) in addition to Foxp3 expression is critical (Ohkura et al., 2012). Thus, nTreg-Me is independent of Foxp3 expression, but necessary for Foxp3^+^ cells to acquire the genome-wide transcriptional profile, stability, and functional characteristics of the Treg lineage (suppressive capability) (Ohkura et al., 2012). Interestingly, *in vitro* induced iTregs lack the nTreg-Me pattern, whilst *in vivo* generated iTreg gradually develop it after TCR stimulation (Ohkura et al., 2012). This difference in stability of Foxp3 expression between tTregs and iTregs could then be attributed to epigenetic differences at the Foxp3 locus, as detailed below. The third, as has been elegantly described recently in the mouse, is that differentiation of both tTregs and iTregs is critically dependent on transcriptional repression of alternate lineages through the expression of the Bach2 TF (Roychoudhuri et al., 2013). Thus, animals deficient in this TF are unable to generate Tregs and succumb to spontaneous autoimmune disease (Roychoudhuri et al., 2013). Interestingly, this TF is also linked to multiple autoimmune diseases in man.

#### *Foxp3* promoter

CpG motifs in the *Foxp3* promoter are basally demethylated in resting Tregs, but partially methylated in conventional naïve CD4^+^ T cells (Kim and Leonard, 2007; Janson et al., 2008). In addition, histones in this region are more highly acetylated in Tregs than in naïve T cells (Mantel et al., 2006; Kim and Leonard, 2007). As a result, the *Foxp3* promoter is more accessible for the binding of TFs, such as NFAT, AP1, STAT5, TIEG1, and Ets1 and 2 [recently described to bind the *Foxp3* promoter (Fayyad-Kazan et al., 2010)] in Tregs than in conventional T cells. In mice, the Foxp3 promoter in conventional T cells remains methylated following TCR activation, albeit at a lower level than at baseline (Janson et al., 2008), and demethylation requires activation in the presence of TGF-β (Kim and Leonard, 2007; Janson et al., 2008); these structural effects limit and promote access for induction of Foxp3 transcription respectively.

#### CNS1 (TGF-β-sensitive enhancer element)

CNS1, an “enhancer” region in the *Foxp3* locus, contains binding sites for NFAT and Smad3 and is in an accessible, histone-acetylated, state in both tTregs and iTregs (Tone et al., 2008) but not in naïve, resting T cells. This area has no CpG motifs, therefore the sole epigenetic modification at this locus is through histone acetylation. RAR and RXR (retinoid X receptor) bind within CNS1 and are responsible for increased histone acetylation, permitting greater Smad3 binding (Xu et al., 2010), thus, explaining some of the direct effects of ATRA in iTreg induction. CNS1 knockout animals demonstrate normal tTreg development, but have impaired iTreg induction (Zheng et al., 2010); therefore, CNS1 is redundant in thymic Treg selection, but is essential for peripheral induction of Tregs, consistent with the role of TGF-β in pTreg generation.

#### CNS2 (“Treg-specific demethylated region”, TSDR)

A third, highly conserved, CpG dinucleotide-rich region in both mouse and human Th cells, termed the “Treg-specific demethylated region” (TSDR), is completely demethylated in nTregs, but methylated in conventional T cells (Baron et al., 2007; Floess et al., 2007). In tTregs, this area also contains acetylated histones (H3Ac and H4Ac) (Floess et al., 2007) and TF binding sites, which in the demethylated state bind Stat5, CREB/ATF (Yao et al., 2007; Nagar et al., 2008), Foxo1, and Foxo3 (Ouyang et al., 2010), which also bind the *Foxp3* promoter (Harada et al., 2010; Ouyang et al., 2010). Interestingly, Foxp3 induction by TGF-β is associated with only partial demethylation of the TSDR, an unstable state that reverses upon restimulation (Floess et al., 2007). Thus, iTregs contain methylated CpGs. The TSDR was initially described as having enhancer activity (Kim and Leonard, 2007). However, given quantitatively similar Foxp3 expression in iTregs and tTregs despite large differences in methylation state at the TSDR, it is unlikely that it acts as an enhancer element; instead, TSDR demethylation appears critical for stable Foxp3 expression (Floess et al., 2007; Nagar et al., 2008; Polansky et al., 2008). Indeed, pharmacological inhibition of DNA methyltransferase-1 (Dnmt-1) in conventional T cells, using the covalent inhibitor 5-azacytidine (5AzadC), followed by activation through the TCR, results in stable expression of Foxp3, in contrast to the transient Foxp3 expression seen with TCR activation alone (Kim and Leonard, 2007; Nagar et al., 2008; Polansky et al., 2008; Josefowicz et al., 2009). Similarly, CNS2-deficient animals have reduced Treg numbers only after 6 months of age (Zheng et al., 2010), suggesting that CNS2 is redundant for expression of Foxp3 but critical for its stable maintenance. Of note, demethylated CNS2 acts as a binding site for Foxp3 in a Runx1- and Cbf-β-dependent manner (Zheng et al., 2010), which may serve as a mechanism for stable Foxp3 expression in mature Tregs. The signals controlling methylation/demethylation at the TSDR are currently unknown, but given the difference between iTregs and tTregs in Foxp3 stability, it is likely that demethylation at this locus is thymically initiated.

#### CNS3 (Pioneer element)

CNS3 contains a DNase I hypersensitive site and is bound by c-Rel, IκB_NS_, and p50, members of the NF-κB family (Zheng et al., 2010; Schuster et al., 2012). Chromatin modifications at this site show permissive marks (H3K9/14Ac, H3K4me2, and H3K4me1) in Tregs, but also mono (H3K4me1) and di-(H3K4me2) methylation in Treg-precursors (CD4^+^CD8^+^ and CD4^+^CD8^-^ thymocytes) (Zheng et al., 2010). As permissive marks are absent at CNS1 and 2 in Treg-precursors, this argues that CNS3 can bind TFs before both CNS1 and 2 during Treg induction and opens the *Foxp3* locus to other TFs, thus acting as a pioneer element (Zheng et al., 2010). Indeed, CNS3^−*/*−^ mice have significantly reduced Treg numbers, but normal per cell levels of Foxp3 in the remaining Tregs, supporting the assertion that CNS3 acts as a pioneer element. The importance of c-Rel binding is highlighted by the profound loss of Tregs seen in mice that are c-Rel deficient (Ruan et al., 2009; Zheng et al., 2010). It is possible that the binding of c-Rel acts as a chromatin opener or, that c-Rel, in co-operation with other TFs, e.g., p65, NFAT, CREB, and Smad3, enhances formation of an enhanceosome at the Foxp3 promoter (Ruan et al., 2009).

### Distinguishing tTregs from iTregs

To date, no single marker has been identified to differentiate tTregs from iTregs and no definitive test to distinguish their *in vivo* functions. The Treg transcriptional profile is dominated by genes induced by cell activation alone (Hill et al., 2007) and has so far not yielded definitive markers to distinguish iTregs from tTregs despite early promise (Gavin et al., 2007; Zheng et al., 2007). Reports that tTregs exclusively express Helios, an Ikaros transcription factory family member (Thornton et al., 2010), have been challenged by the demonstration that Helios is induced during T cell activation and proliferation and then down-regulated (Akimova et al., 2011). Thus, expression of Helios cannot reliably differentiate iTregs from tTregs. Likewise, although TSDR demethylation could in theory distinguish Tregs that have received thymic induction from those induced in the periphery, *in vivo* generated iTregs can also efficiently demethylate the TSDR if given sufficient time (in this case, 6 weeks) (Polansky et al., 2008). Very recently, neuropilin-1 (Nrp-1) has been described by two groups as differentially expressed in murine tTregs and pTregs, being poorly expressed in the latter (Weiss et al., 2012; Yadav et al., 2012). Nrp-1 is a cell surface molecule mediating prolonged interactions between Tregs and DCs (Sarris et al., 2008), a receptor for TGF-β (Glinka and Prud’Homme, 2008) and vascular endothelial growth factor (Ferrara et al., 2003), which has previously been proposed as a Treg marker (Bruder et al., 2004; Hansen et al., 2012). Of interest, the lowest Nrp-1 expression was seen in *in vivo* generated pTregs compared to *in vitro* generated iTregs, presumably reflecting positive regulation of Nrp-1 by high dose TGF-β *in vitro* (Weiss et al., 2012). These observations have not yet been replicated in human Tregs, although Nrp-1^+^ Tregs have been identified in inflamed synovial fluid of patients with rheumatoid arthritis (E et al., 2012).

### tTreg and iTreg function

Tregs suppress target cells through a number of inhibitory mechanisms, including cell–cell contact-dependent inhibition (Takahashi et al., 1998; Thornton and Shevach, 1998; Ng et al., 2001), secretion of inhibitory cytokines (Powrie et al., 1996; Asseman et al., 1999; Belkaid et al., 2002; Maloy et al., 2003; Collison et al., 2007), cytolysis of target cells (Gondek et al., 2005; Cao et al., 2007), metabolic disruption (Deaglio et al., 2007), modulation of APC function (DiPaolo et al., 2007; Puccetti and Grohmann, 2007), and competition for environmental IL-2 (Pandiyan et al., 2007). Such redundancy suggests that the mode of suppression may be context dependent and directed by the degree and mode of inflammation. While details of these mechanisms falls outside the scope of this review, it is noteworthy that they are not mutually exclusive, and while not necessarily limited to a single “delivery system”, are mostly compatible with a cell-to-cell contact deployment package. For example, Tregs can deliver suppressive factors like cyclic adenosine monophosphate (cAMP) into conventional T-cells via gap junctions (Bopp et al., 2007), they can modulate APC function through membrane-bound suppressive TGF-β (Nakamura et al., 2001), through negative signaling by cytotoxic T-lymphocyte antigen-4 (CTLA-4) (Read et al., 2006) or lymphocyte-activation gene 3 (LAG3) (Huang et al., 2004).

So far, no distinct functional differences have been conclusively demonstrated between tTregs and iTregs, suggesting that the mechanistic repertoire of Treg function is specified by lineage and not mode of induction. Indeed, iTregs are as potent as tTregs in protecting from autoimmune diseases by preventing the antigen-presenting capacity of DCs to autoreactive Teffs (DiPaolo et al., 2007). As only a small proportion of the transcriptional profile of Tregs can be explained by expression of Foxp3, and the majority by T cell activation and survival signals (Hill et al., 2007), it is not surprising perhaps that function is also lineage and not induction-specific. As argued above, it is generally accepted that tTregs function to prevent the development of autoimmune diseases and that iTregs limit inflammation to neo-antigens, such as bowel commensal. iTregs can clearly be generated and are essential and sufficient to mediate oral tolerance in response to dietary antigens in animals devoid of tTregs (Mucida et al., 2005; Curotto de Lafaille et al., 2008). Although these experiments have been conducted under highly non-physiological conditions, the same mechanisms of iTreg induction in the periphery may explain the persistence of alternative neo-antigens, such as pathogenic organisms (Robertson and Hasenkrug, 2006; Wohlfert and Belkaid, 2008) or neoplastic cells (Zou, 2006).

### Treg plasticity

Emerging concepts of mammalian Th cell polarization have recently challenged traditional models of terminal differentiation, suggesting that Th lineage commitment is not as irreversible as previously thought and that lineage reprograming to alternate lineages can be achieved through the expression of key TFs and appropriate epigenetic modifications in lineage-specifying genes. For in-depth reviews, please see (O’Shea and Paul, 2010; Hirahara et al., 2011; Nakayamada et al., 2012). Several reports in the literature suggest that Tregs retain significant plasticity, with the capacity to express TFs and signature cytokines, particularly, of Th1 (Koch et al., 2009) and Th17 (Koenen et al., 2008; Beriou et al., 2009; Afzali et al., 2010) cells.

The ability of Tregs to express key TFs of alternate lineages may license them to efficiently regulate inflammation generated by those Th lineages. For example, T-bet expression by Tregs induces CXCR3 expression, licensing Treg trafficking to sites of Th1-mediated inflammation to control Th1 cells (Koch et al., 2009). Likewise, expression of IFN-γ by Tregs may be a surrogate marker for T-bet expression and licensing for suppression of Th1 inflammation (Feng et al., 2011). Expression of interferon regulatory factor-4 (IRF4), a TF essential for Th2 (Rengarajan et al., 2002) and Th17 (Brüstle et al., 2007) cell differentiation directs Tregs to selectively regulate Th2 responses (Zheng et al., 2009). Selective ablation of Stat3, critically required for Th17 differentiation (Figure 2), in Tregs results in uncontrolled Th17-dependent responses (Chaudhry et al., 2009).

On the other hand, plasticity in Tregs may indicate a potential to assume an effector phenotype and to contribute to inflammation (Zhou et al., 2009b). In particular, as Treg and Th17 differentiation from naïve Thp are reciprocally linked (Bettelli et al., 2006; Mangan et al., 2006; Veldhoen et al., 2006; De Jong et al., 2010) (see Figure 2) and the two lineages have opposing functions (Afzali et al., 2007), lineage reprograming from one to the other could have significant implications for the development of autoimmune diseases and for programs of Treg-based cell therapy in humans. It is certainly possible that there may be a threshold of expression and/or activation of Th-specific TFs in Foxp3^+^ Tregs allowing them to act either as lineage-specific regulators or contributors to effector responses. There remains still considerable controversy regarding Treg plasticity and lineage reprograming as even complex, and elegant, fate-mapping murine models (Zhou et al., 2009b; Hori, 2010, 2011; Rubtsov et al., 2010) have produced divergent results.

Mechanistically, the epigenome of many “terminally differentiated” Th cells shows considerable flexibility in accessibility of genes of alternate lineages to TFs. This is elegantly described in the study of Wei et al. (2009) showing a rather flexible signature of genome-wide H3K4me3 and H3K27me3 maps of naïve, Th1, Th2, Th17, iTreg, and tTreg cells. In this study, the methylation of loci for signature cytokines conformed broadly to that expected from lineage commitment; however, the epigenome of “master” TFs showed significant flexibility, presenting both permissive and repressive modifications in the various Th subsets, including bivalent epigenetic states. This suggests that the overall balance of epigenetic state determines cell differentiation and that bivalent modifications might allow specific lineage regulator gene loci to be activated under different polarizing conditions, thus reprograming Th cells into other lineages. For example, tTregs and iTregs both have repressive H3K27me3 marks at the *Il17a* locus. This is in contrast to permissive H3K4me3 at the *Rorc* locus in iTregs and bivalent chromatin at this locus in tTregs (Wei et al., 2009), potentially permitting co-expression of Foxp3 and RORγt after culture under Th17 polarizing conditions (Xu et al., 2007; Yang et al., 2008). Signals from the micro-environment are then clearly key to lineage stability. While much focus has been on the local cytokine cytokine milieu, recent data has also highlighted the role of local complement components, notably the anaphylatoxins C3a and C5a, which can signal through cognate receptors on Tregs to down-regulate Foxp3 expression by activating Akt (Kwan et al., 2013).

Given the difficulty in distinguishing pTregs from tTreg in a healthy host, no definitive experiment has yet conclusively shown a difference in Th17 plasticity between tTregs and iTregs *in vivo* (Zhou et al., 2009b). Human data is also inconclusive; while it appears that Th17 plasticity is restricted to a population of suppressive memory Tregs expressing the lectin receptor CD161 (Afzali et al., 2013; Pesenacker et al., 2013), divergent reports suggest that these cells are thymically derived (predominantly demethylated TSDR, Pesenacker et al., 2013) and peripherally induced [low *Helios* expression, virtual absence from umbilical cord blood and CD45RA^-^ phenotype (Ayyoub et al., 2009; Afzali et al., 2013)].

### miRNA and Tregs

Gene transcription events are also heavily influenced by microRNAs (miRNAs or miRs) and recent evidence supports the role of this class of molecules in Treg biology. miRNAs are an evolutionarily conserved class of pleiotropically acting small endogenous RNAs, about 23 nucleotides long, that play important gene-regulatory roles by pairing to the mRNAs of protein-coding genes to direct their post-transcriptional repression. miRNAs are predominantly transcribed by RNA polymerase II, which produces a primary transcript containing the mature miRNA sequence and a varying amount of flanking region (Lee et al., 2004). Two nucleases then process the miRNA: the first one, Drosha, cleaves the primary miRNA into a precursor miRNA (Han et al., 2006) that is exported from the nucleus by exportin 5 (Yi et al., 2003); after reaching the cytoplasm, the precursor miRNA is further processed by the other nuclease, Dicer, and is loaded into the RNA-induced silencing complex (RISC) (Chendrimada et al., 2007). Finally, a specific single strand of the miRNA duplex is selected as a guide to direct sequence-specific targeting of mRNA 3’ untranslated regions (UTRs) by RISC (Bartel, 2009). In mammalian cells, miRNAs silence genes mainly through binding of target mRNA leading to degradation of the mRNA; however, another mechanism of repression at a translational level has been reported, showing that miRNAs can inhibit either the initiation or the elongation stages of protein translation (reviewed in Pillai et al., 2007; Lodish et al., 2008). Interestingly, given the short sequence and non-stringent binding to target sequence, abiding to a Watson–Crick match, an individual miRNA is capable of regulating dozens of distinct mRNAs (Bartel, 2009). For a general review on miRNAs, please see (Chen and Rajewsky, 2007).

MicroRNAs have been implicated as fundamental regulators of post-transcriptional programs and play a role in T-lymphocyte development, differentiation, and effector functions since they are differentially expressed, both spatially and temporally, in many types of immune cells (Lykken and Li, 2010). MicroRNA appear critical for the Treg phenotype, as conditional knockout of *Dicer* in CD4 cells (*CD4*^CreDicer^^Δ*/*Δ^ animals) results in substantial depletion of tTregs and inhibits induction of Foxp3 in naïve CD4 T-cells by TGF-β (Cobb et al., 2006). These mice develop spontaneous autoimmune disease from about 3–4 months of age, in contrast to conditional knockout of *Dicer* in Foxp3^+^ cells (*Foxp3*^CreDicer^^fl/fl^), which results in spontaneous autoimmune disease that is fatal by 4 weeks of age (Liston et al., 2008a; Zhou et al., 2008b). In the latter model, Foxp3 expression is unstable and Tregs revert to an effector phenotype producing IL-4 and IFN-γ as part of the disease (Zhou et al., 2008b). Likewise, conditional disruption of *Drosha* in CD4 cells produces a very similar phenotype (Chong et al., 2008). *Dicer* and *Drosha* knockout, however, results in ablation of not only canonical miRNAs, but also that of other small cellular RNA species (e.g., siRNAs and shRNAs). That the phenotype of mice with ablated *Dgcr8*, an RNA-binding protein required in the processing of canonical miRNAs (Babiarz et al., 2008), resembles that of the Dicer deficient mice (Jeker et al., 2013) establishes that miRNAs are critical for normal Treg development in the thymus and the periphery and they are essential for normal Treg function. Conversely, Foxp3 also contributes to the miRNA signature of Tregs (Cobb et al., 2006; Rouas et al., 2009).

**Figure 4:**
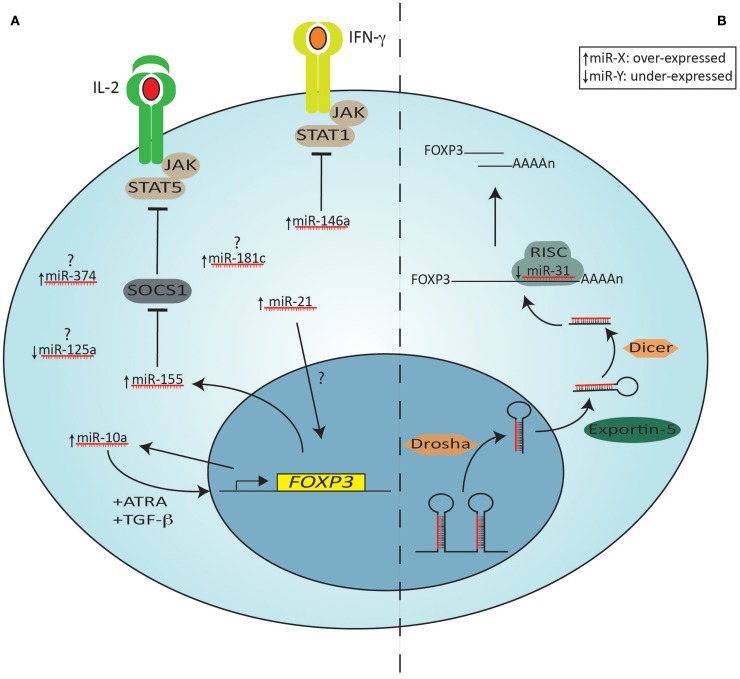
**The miRNA machinery and miRNAs differentially expressed in Tregs compared to other CD4^+^ T cells**. Shown on the left **(A)** are specific miRNAs that are over (arrow pointing up) or under (arrow pointing down)-expressed in Tregs compared to other Th cells. miRNAs that are over-expressed (miR-374, miR-181c) or under-expressed (miR-125a) without a known target or function in Tregs are indicated with a question mark. Some miRNAs are induced by Foxp3, leading to either down-regulation of a specific target (e.g., miR-155 repressing SOCS1) or inducing positive feedback on *Foxp3* expression (miR-10a, in combination with ATRA and TGF-β). miR-21 is also an indirect positive regulator of *Foxp3*, but its mechanism of action is still unknown. Shown on the right **(B)** is the miRNA processing and targeting machinery, depicting miR-31, an under-expressed microRNA in Tregs, which targets the 3′ UTR of *Foxp3* mRNA. The primary miRNA transcript is first processed in the nucleus by Drosha. The precursor is then exported from the nucleus by Exportin 5 and in the cytoplasm a second nuclease, Dicer, generates a double stranded miRNA. The functional strand is subsequently selectively loaded onto RISC. Binding of the mature miRNA to the 3′ UTRs of the target mRNA leads to its degradation. JAK = Janus kinase; STAT = Signal Transducer and Activator of Transcription; SOCS = suppressor of cytokine signaling proteins; RISC = RNA-induced silencing complex (RISC); UTR = untranslated region.

Of the miRNAs that are important for Treg function, only a few are known and the exact function(s) of these are still largely unknown. As a single miRNA can regulate potentially thousands of genes, small differences in miRNA profiles can have profound effects on T cell function. The miRNA machinery and miRNAs that are differentially expressed in Tregs, including those known to be direct Foxp3 targets are shown in Figure 4.

Amongst these, miR-31 is under-expressed in human Tregs while miR-21 is over-expressed in both human and mouse Tregs. Using lentiviral transduction studies, it can readily be seen that miR-31 and miR-21 have opposing effects on FOXP3/Foxp3 expression. Whilst miR-31 negatively regulates FOXP3 (it has a direct binding site in the 3′UTR of *FOXP3* mRNA), mir-21 positively regulates FOXP3/Foxp3 in an indirect, but still not fully elucidated, manner (Rouas et al., 2009). Of interest, histone deacetylase inhibition using valproate reduces miR-31 and increases mir-21 as well as FOXP3 expression in human Teffs to levels seen in Tregs (Fayyad-Kazan et al., 2010). This change in miRNA profile is independent of the change in FOXP3 expression (Fayyad-Kazan et al., 2010).

MicroRNA-155, which has previously been studied in T and B cell biology (Baltimore et al., 2008), is a direct Foxp3 target (Marson et al., 2007; Zheng et al., 2007; Liston et al., 2008a), and highly expressed in Tregs. Mir-155 targets suppressor of cytokine signaling 1 (Socs1), enhancing Stat5 signaling. As a result, deletion of miR-155 results in limited Stat5 signaling, attenuating IL-2 signaling, manifesting as reduced thymic and peripheral Treg numbers (Lu et al., 2009). It may also target Foxo3a, albeit in a Treg cell line (Yamamoto et al., 2011).

Mir-146a is another microRNA prevalently expressed in Tregs that targets Stat1; deletion of mir-146a in Tregs causes a severe autoimmune phenotype akin to Dicer knockout animals, characterized by increased numbers of poorly functional Foxp3^+^ Tregs in the periphery (Lu et al., 2010). As thymic Treg numbers are unaltered, it is likely that the biological role of mir-146a is preferentially to regulate Treg gene expression in the periphery. Indeed, not only do *miR-146a*^−*/*−^ Tregs fail to control Teffs in the periphery, but they also gain Th1-like properties, such as secretion of IFN-γ (Lu et al., 2010), as a result of failure to regulate Stat1 signaling (Tang et al., 2009; Lu et al., 2010).

None of miR-21, miR-31, miR-155 nor miR-146a have been shown to regulate gene expression preferentially in tTregs compared to iTregs or vice versa. Mir-10a, on the other hand, is preferentially expressed in tTregs, but poorly expressed in iTregs induced with TGF-β without ATRA (Jeker et al., 2012; Takahashi et al., 2012). Of note, expression of miR-10a is lowest in Tregs from animals prone to autoimmune disease, such as non-obese diabetic (NOD) mice, and in Tregs with unstable Foxp3 expression (Jeker et al., 2012). miR-10a expression in Tregs that lose Foxp3 expression is the same as in Teffs (Jeker et al., 2012). miR-10a is functionally linked to stabilization of Foxp3 expression (Jeker et al., 2012) and targets the transcriptional repressor Bcl-6 and corepressor Ncor2 to limit conversion of iTregs to Tfh (Takahashi et al., 2012). It also fine-tunes Thp fate decisions between iTreg and Th17 (Takahashi et al., 2012).

Thus, these studies show a defined requirement of miRNAs for the differentiation and suppressive function of Treg cells as well as their lineage stability. Differential expression of miRNAs in tTregs and iTregs could reflect divergent pathways of differentiation, functional properties or lineage stability.

## Other, Foxp3^−^, T Cells with Regulatory Function

In addition to Tregs, a number of other inducible T cells have been described with regulatory properties. These include members of the CD4^+^ (Th3, Tr1, and iTr35) and CD8^+^ (CD8^+^CD28^−^) families. Amongst these, one of the most controversial is the T helper 3 (Th3) subset. This subset was described as an unusual Th2-like regulatory subset, which secretes TGF-β, derived from orally tolerized animals induced by mucosal stimulation with antigen (Chen et al., 1994). Thus, Th3 cells could be induced through cognate stimulation of CD4^+^ Thp by APC together with CD86 co-stimulation, particularly in the presence of TGF-β and IL-4 (Inobe et al., 1998; Seder et al., 1998; Weiner, 2001). Further growth and division of Th3 cells was dependent on IL-4 and TGF-β rather than IL-2, and some Th3 clones produced IL-4 and/or IL-10 together with TGF-β. Th3-mediated suppression, for the maintenance of oral tolerance, was described as mediated by TGF-β, secreted in response to CTLA-4 ligation (Chen et al., 1998). There is, thus, a degree of similarity between Th3 cells and iTregs given their peripheral (TGF-β-enhanced) induction, mucosal location and TGF-β-dependent function. The lack of iTreg- or Th3-specific markers effectively ensures that the two populations cannot at present be distinguished as disparate.

Type 1 regulatory T cells (Tr1) cells are Foxp3^-^ regulatory T cells that are induced in the periphery in a TCR-dependent and antigen-specific manner through either repeated stimulation with antigen or encounter of antigen in the context of immature DCs (Jonuleit et al., 2000; Dhodapkar et al., 2001) or IL-10 (Groux et al., 1997), with or without IFN-α (Levings et al., 2001). Thus, potent Tr1 induction can be achieved through stimulation of human T cells with a subset of IL-10 producing tolerogenic DCs (DC-10) (Gregori et al., 2010). IL-10 produced by DC-10 stimulates HLA-G expression on target Thp; HLA-G subsequently binds ILT4 (immunoglobulin-like transcript 4) on the DC-10 to augment Tr1 induction (Gregori et al., 2010). Intriguingly, engagement of complement receptor CD46 induces IL-10 producing T-cells phenotypically similar to Tr1 cells (Kemper et al., 2003). Since such complement engagement occurs *in vivo* (Le Friec et al., 2012) and *in vitro* (Cardone et al., 2010) to induce Th1 cells before switch to a Tr1 phenotype, an interesting possibility remains that a “Tr1 phenotype” could also represent a final common pathway of activated T cells that have gone through an inflammatory phase and have entered a self-regulatory, IL-10 producing, phase required for wound healing and tissue repair. Indeed, this would comply with the fact that Th1 cells, Th2 cells, and Th17 can all produce IL-10, as is further discussed in an accompanying article in this series. Interestingly, ATRA inhibits IL-10 production, in contrast to augmentation of Foxp3 (Maynard et al., 2009); thus it is possible that induction of Tr1 cells and iTreg in an ATRA-containing environment are to an extent mutually exclusive.

Tr1 cells are anergic, proliferate poorly to antigen, produce little IL-2 or IL-4, but suppress through production of IL-10 and TGF-β (Groux et al., 1997). In addition, they secrete IFN-γ and IL-5; thus their cytokine profile is distinct from Th1, Th2 and classical Tregs (Groux et al., 1997). Although they do not constitutively express Foxp3 (Vieira et al., 2004), and can be generated in *FOXP3* mutant patients with IPEX syndrome (Passerini et al., 2011), they are able to mediate their suppressive function through multiple mechanisms, such as engagement of CTLA-4 and Programed cell death 1 (PD1) (Akdis et al., 2004; Meiler et al., 2008), metabolic disruption through CD39 and CD73 (Mandapathil et al., 2010), and cytolysis of APCs through release of granzyme B and perforin (Magnani et al., 2011). Thus, they share functions in common with Foxp3^+^ Tregs. Until now, no reliable markers could successfully distinguish Tr1 cells from other IL-10 producing T cells. However, the recent description of co-expression of integrin α-subunit CD49b and lymphocyte-activation gene (LAG)-3 as identifiers of human and mouse Tr1 cells (Gagliani et al., 2013), will allow further specific characterization of Tr1 cell genesis and function as well as its relation to other IL-10 producing T cells.

**Figure 5:**
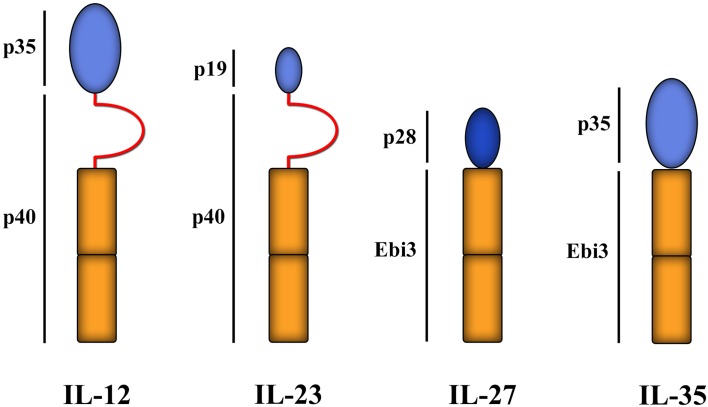
**IL-12 family of cytokines**. Members of the structurally related IL-12 family of cytokines all comprise of a helical subunit (depicted as blue ovals) and a cytokine receptor homology domain (depicted as orange rectangles) with or without an intervening immunoglobulin-like domain (red semi-circles). Thus far, four family members have been identified: IL-12, IL-23, IL-27, and IL-35. Ebi3 = Epstein–Barr-Virus-induced molecule 1.

IL-35 is a member of the IL-12 cytokine family (Figure 5) with inhibitory functions. It was originally described in murine cells as a heterodimeric suppressive cytokine secreted from Foxp3^+^ Tregs [the Ebi gene is a downstream target of Foxp3 (Collison et al., 2007)], without which the suppressive function of Tregs was significantly reduced, rendering Tregs incapable of controlling experimental inflammatory bowel disease (Collison et al., 2007). Secretion of IL-35 by Tregs is increased by co-culture with Teffs, subsequently enabling them to suppress Teffs separated by a semi-permeable membrane (Collison et al., 2009). In both man and mouse, IL-35 can induce the development of T cells that secrete IL-35, but not TGF-β or IL-10, and can then mediate suppression in an IL-35-dependent manner. These induced regulatory T cells have been termed iTr35 (Collison et al., 2010; Chaturvedi et al., 2011). iTr35 cells are hyporesponsive to restimulation and, like Tr1 cells (see below), don’t express the TF Foxp3. Moreover, they can be induced from *Foxp3*^−*/*−^ murine Thp (Collison et al., 2010), showing that Foxp3 is neither required for their induction nor for their function. Of note, however, iTr35 cells have a gene transcriptional profile that is very similar to non-suppressive Teffs activated without IL-35 (though very different to Tregs) (Collison et al., 2010), suggesting that the induction of iTr35 cells, as with iTregs, is dominated by signals that are generic to T cell activation/survival and requires only modest transcriptional changes induced by IL-35. Although the exact role of IL-35 and iTr35 cells in immune physiology is not known, ectopic expression of IL-35 on pancreatic β-cells can protect against experimental autoimmune diabetes (Bettini et al., 2012) and can be expressed by other immune cells, such as CD8^+^CTLA-4^+^ T cells that can suppress tumor (prostate)-specific Teff responses (Olson et al., 2012). The induction of iTr35 cells by neighboring cells producing IL-35, such as Foxp3^+^ Tregs, may be important in providing at least partial explanations for the phenomenon of infectious tolerance (Waldmann et al., 2006), which hypothetically could be a key component in the success or failure of Treg-based programs of cell therapy.

Briefly, T-lymphocytes with regulatory functions are not only limited to the CD4^+^ population, but include some CD8^+^ populations as well. Gilliet and Liu, for instance, demonstrated that stimulation of naïve CD8^+^ T-cells with CD40 ligand-activated plasmacytoid DCs induced, in an IL-10-dependent manner, poorly proliferative CD8^+^ T-cells. These cells produced significant amounts of IL-10, low IFN-γ, no IL-4, IL-5, nor TGF-β, and suppressed CD8^+^ alloresponses through IL-10 (Gilliet and Liu, 2002). Likewise, repeated stimulation of CD8^+^ T cells with antigen can generate suppressive CD8^+^CD28^−^ T cells (Jiang et al., 1998) that show significant overlap in molecular signature with Tregs (Foxp3, GITR, CTLA-4, CD25 for example) (Scotto et al., 2004). The loss of CD28 on CD8 cells, through repeated stimulation, is a well recognized phenomenon and occurs physiologically during aging (Weng et al., 2009) and correlates with poorer responses to vaccination (Saurwein-Teissl et al., 2002). These cells may have a variety of suppressive mechanisms that include inhibition of co-stimulatory molecules on T cells (Ciubotariu et al., 1998) or DCs (Li et al., 1999).

## Cell-Based Therapy Using Tregs

The critical role played by Tregs in maintaining peripheral tolerance to self-antigens, thereby controlling autoimmune diseases, reveals the clinical potential of these cells, which can find extensive application to induce transplant tolerance (Wood and Sakaguchi, 2003; Hippen et al., 2011; Issa et al., 2011; Lombardi et al., 2011; Blazar et al., 2012; Tang et al., 2012). In this context, emerging data from animal models reveals that adoptive transfer of Tregs could ameliorate autoimmune diseases, graft-versus-host disease (GvHD) (Blazar et al., 2012) and also induce tolerance to solid organ transplants (Issa et al., 2011). These findings suggest that clinical therapy with human Tregs represents a promising strategy for treatment of autoimmune diseases or for induction of transplantation tolerance.

In solid organ transplant recipients, allo-reactive Teffs in the immune repertoire outnumber Tregs, causing inflammation and leading to graft rejection. So far, most, if not all, therapies aimed at preventing transplant rejection have targeted Teffs. However, another approach, artificially increasing Treg number to regulate Teffs (Figure 1), also has the potential to promote tolerance and facilitate graft survival (Safinia et al., 2013). This is supported by evidence showing that prolonged organ engraftment is essential to induce and expand allo-antigen-specific Tregs, favoring long-term acceptance (Hamano et al., 1996) and data that show better transplant outcomes when organs are infiltrated with greater numbers of Tregs.

There are effectively three strategies for using Tregs as therapeutic agents in humans. The first is introduction of freshly isolated donor Tregs into lymphopaenic hosts, an approach most attractive in the prevention of GvHD post-bone marrow transplantation (Di Ianni et al., 2011). The lymphopaenic environment supports expansion of infused Tregs *in vivo* and does not cause over-immunosuppression. Indeed, similar experiments in mice have shown that the animals are immunologically intact and able to respond to vaccination (Gaidot et al., 2011) and to control influenza virus infections (Bushell et al., 2005) using this approach.

The second approach involves the *in vitro* expansion of Tregs prior to infusion, a pre-requisite for infusion of large numbers of Tregs, since their numbers in the peripheral circulation are low. Using polyclonal activation and high doses of IL-2 to expand Tregs could provide the necessary number for therapeutic efficacy. However, intensive expansion protocols could compromise purity of Tregs at the end of the culture protocol. These limitations may be in part due to the presence of “contaminating” Teffs within bead-separated Treg preparations; however the capacity for conversion of human Tregs into IL-17-producing cells has also been well demonstrated (see above). To this aim, the application of tolerogenic approaches to both enhance Treg expansion *in vitro* and stabilize their suppressive phenotype has been investigated in recent years. Rapamycin, an mTOR kinase inhibitor, for example, selectively promotes expansion of suppressive human Tregs (Battaglia et al., 2006; Scotta et al., 2012). Likewise, culture of Tregs *in vitro* in the presence of ATRA also supports expansion of functional FOXP3^+^ human Tregs (Scotta et al., 2012). In contrast, only Treg cultures propagated in the absence of Rapamycin contain CD161^+^ Tregs, the precursor population of IL-17-producing Tregs (Tresoldi et al., 2011; Scotta et al., 2012). Thus, culture of Tregs with a combination of Rapamycin and clinically acceptable retinoic acid-related molecules may be a viable option to generate large numbers of suppressive and stable Tregs with limited IL-17 potential (Golovina et al., 2011; Scotta et al., 2012). However, among the first Treg-based cell therapy trials in humans (for the treatment of GvHD and type 1 diabetes mellitus respectively), two have used no drug supplementation (except for IL-2) during *ex vivo* expansion of Tregs and neither has reported unexpected side effects nor paradoxical exacerbation of disease in patients (Trzonkowski et al., 2009; Marek-Trzonkowska et al., 2012).

Neither of the first two approaches, however, makes a distinction between tTregs and iTregs as the starting population. Indeed, culture of contaminating Teffs in the presence of Rapamycin or ATRA during Treg expansion would support the development of iTregs, as discussed above. Thus, the third approach for Treg-based therapy is the induction of iTregs *in vivo*. As previously discussed, induction of Tregs in the periphery, whether Foxp3^+^ or Foxp3^-^ can be achieved through a variety of means. Therapeutic options can therefore include administration of tolerogenic DCs that support the *in vivo* development of both iTregs and Tr1 cells (Naranjo-Gómez et al., 2011; Boks et al., 2012), injection of *in vitro* expanded Tr1 cells (Brun et al., 2009; Desreumaux et al., 2012) or the introduction of regulatory macrophages (Mregs – not discussed here).

Although these data provide only the earliest evidence for the clinical application of Tregs in cell therapy, a strategy to use these approaches in solid organ transplantation is under way. The ONE Study, for instance, is a multi-center phase I/II study, funded by the European Union FP7 program, investigating the safety of infusing *ex vivo* generated/expanded Tregs, Tr1 cells, Mregs and tolerogenic DC into kidney transplanted recipients. Altogether, about 200 patients will be enrolled in this clinical trial and, importantly, every center will use the same immunosuppressive protocol for both cell therapy as well as control arms. All patients will be extensively monitored, to obtain data regarding safety, pharmacodynamics, and efficacy of cell therapy, providing an extensive data set for future clinical trials.

## Conclusion

Immunological homeostasis is a delicate balance in which both excessive and suboptimal responses can lead to pathological states. CD4^+^ T cells can differentiate to different Th subsets and promote either an inflammatory response (Th1, Th2, and Th17) or a regulatory one (Tregs). Are then Tregs always beneficial for the optimal resolution of homeostatic challenges? As always, when considering immunological homeostasis, the situation is similar to “Goldilocks and the three bears.” While Tregs are essential to prevent autoimmune disease (Asano et al., 1996) and minimize inflammatory immune responses against dietary antigens and commensal flora (Izcue et al., 2006), excessive Treg responses may facilitate tumor growth and chronic infections by limiting anti-tumor (Shimizu et al., 1999) or anti-pathogen responses (Sakaguchi, 2005). Thus, Tregs function must be tightly regulated to ensure responses are appropriate for each pathological scenario (reviewed in Belkaid, 2007).

Regulatory T cells are both centrally derived and peripherally induced and include both Foxp3^+^ and Foxp3^−^ populations of cells. An understanding of the mechanisms of Treg induction, suppressive function and lineage stability is key to unraveling the causes underlying development of autoimmune diseases and the design of studies employing Tregs as therapeutic tools in the clinic. Important questions include which regulatory population(s) we should use, whether/how they should be expanded *in vitro* or induced *in vivo*, what role infectious tolerance will play, whether Treg plasticity will pose a significant problem and whether the epigenetic/miRNA profile should/could be exploited. On the other hand, lineage plasticity could in theory allow the conversion of effector Th1 and Th17 cells into functioning Tregs in a therapeutic manner. Increasing numbers of clinical trials are focusing on the use of Tregs in a clinical setting, suggesting that Treg-based therapy is considered as both a feasible and acceptable approach to treat inflammatory diseases, offering an alternative to standard pharmacological care. Answers to the questions posed here should, therefore, be forthcoming.

## Conflict of Interest Statement

The authors declare that the research was conducted in the absence of any commercial or financial relationships that could be construed as a potential conflict of interest.
